# Synchronous Primary Metastatic Infra-Mammary Accessory Breast Cancer and Ipsilateral Breast Cancer: An Extremely Rare Case Report

**DOI:** 10.3390/diagnostics14232699

**Published:** 2024-11-29

**Authors:** Marius Preda, Nilima Rajpal Kundnani, Roxana Buzas, Sorin Dema, Adrian Carabineanu, Codruta Dana Miclaus, Razvan Ilina, Octavian Marius Cretu, Alexandru Blidisel

**Affiliations:** 1 Second Discipline of Surgical Semiology, Department IX-Surgery-1, “Victor Babeș” University of Medicine and Pharmacy, 300041 Timișoara, Romania; 2 Second Clinic of General Surgery and Surgical Oncology, Emergency Clinical Municipal Hospital, 300079 Timișoara, Romania; 3 Breast Surgery Research Center, “Victor Babeș” University of Medicine and Pharmacy, 300041 Timișoara, Romania; 4 Discipline of Internal Medicine and Ambulatory Care, Prevention and Cardiovascular Recovery, Department VI-Cardiology, “Victor Babes” University of Medicine and Pharmacy, 300041 Timișoara, Romania; 5 Research Centre of Timisoara Institute of Cardiovascular Diseases, “Victor Babeș” University of Medicine and Pharmacy, 300041 Timișoara, Romania; 6 Department of Internal Medicine I-Medical Semiotics I, Centre for Advanced Research in Cardiovascular Pathology and Haemostasis, “Victor Babeș” University of Medicine and Pharmacy, EftimieMurgu Sq. No. 2, 300041 Timișoara, Romania; 7 Discipline of Oncology, Department IX-Surgery-1, “Victor Babeș” University of Medicine and Pharmacy, Eftimie Murgu Sq. No. 2, 300041 Timișoara, Romania; 8 Clinic of Surgical Semiotics and Thoracic Surgery-1, Department IX-Surgery-1, “Victor Babes” University of Medicine and Pharmacy, 300041 Timișoara, Romania; 9 Center for Hepato-Biliary-Pancreatic Surgery (CHBP), “Victor Babeș” University of Medicine and Pharmacy, 300041 Timișoara, Romania

**Keywords:** accessory breast cancer, accessory breast tissue (ABT), infra-mammary region (IMR), multi-modal approach, metastatic breast carcinoma, neoadjuvant chemotherapy, adjuvant radiotherapy

## Abstract

**Background:** Accessory breast cancer cases are rarely reported in the literature. Of the reported cases, the predominantly available ones are those localized in the axillary region. **Methods:** We present here a very rare case of metastatic accessory breast cancer. It was located in the infra-mammary region (IMR). IMR accessory breast cancer is a rare form of breast cancer. Although ectopic nipples are occasionally found in the IMR, because of the lack of ductal tissue malignant changes, they are rare. **Results:** In our case, the primary tumor was localized in the congenital accessory breast tissue (ABT). It was recognized as invasive lobular accessory breast cancer cT3N1M0 with a second NST carcinoma, cT2N0M0, Stage IIA, in the ipsilateral breast. A multi-modal approach was applied. Adjuvant chemotherapy was carried out with epirubicin, cyclophosphamide, and paclitaxel, with post-chemotherapy ultrasound followed by right radical mastectomy. Adjuvant radiotherapy was given to our patient in the form of 25 fractions of 50 GY for 25 days, followed by hormonal treatment with Letrozole, 2.5 mg/day, to be continued for 5 years. **Conclusions:** our case demonstrates that since it is rare to find accessory breast cancer in the infra-mammary region, early identification and management with a multi-modal approach can lead to a successful patient outcome.

## 1. Introduction

In women, the most common cancer and the leading cause of cancer-associated death is breast cancer [1]. In 2024, an estimated 310,720 new cases of invasive breast cancer and 56,500 new cases of non-invasive (in situ) breast cancer may be diagnosed in women in the U.S. [2,3].

Accessory breast tissue (ABT) may comprise any cutaneous or subcutaneous tissue, apart from the breast tissue, anywhere along the milk line from the axilla to groin [4,5,6]. ABT may comprise the additional nipple, the complete accessory breast, or the aberrant breast tissue devoid of an areola and/or a nipple [7]. In other words, ABT can be any combination of the breast tissue, the areola, and the nipple. The origin of ABT is based on the failure of involution of the embryologic mammary ridge, also known as the milk line. The ABT is also subject to hormonal influences during puberty, pregnancy, lactation, and menopause in the same manner as the native breast tissue [8,9]. All diseases that can affect the breast can also occur in the accessory breast tissue and this includes benign fibrocystic changes, mastitis, fibroepithelial lesions, ductal or lobular hyperplasia, and malignancy [9,10].

Unfamiliarity with infra-mammary ABT may often delay the diagnosis or suspicion of accessory breast cancer. The absence of a nipple or areola in the ABT further becomes a barrier to the timely detection. Ectopic nipples may be found occasionally in the infra-mammary region (IMR). Since ectopic nipples lack ductal tissue, they preclude the possibility of malignant changes. Hence, IMR accessory breast cancer remains a rare form of breast cancer. Primary carcinomas of ABT are reportedly very rare, with the axilla being their commonest site [11].

The management of accessory breast cancer follows the same procedure as native breast cancer. The approach of treatment is guided by the immunohistochemical profile, staging and overall clinical scenario. It involves surgical resection, chemotherapy, radiotherapy, hormonal therapy, targeted therapy, as well as immunotherapy. Innovative modalities of management such as vaccines, gene therapy, T cell receptor therapy, and chimeric antigen receptor T (CAR-T) therapy are also rapidly evolving [12]. In early invasive breast cancer, the importance of breast conservative surgery and mastectomy is well known, and this may or may not follow reconstruction [12].

We aim to present a very rare case of metastatic accessory breast cancer located in the infra-mammary region.

## 2. Case Description

### 2.1. History

Our patient was a post-menopausal 53-year-old woman. She had noticed a lump in her right IMR in the past 4 months by breast self-examination, which was rapidly growing over the past 2 months. Then, she primarily consulted elsewhere, and 14 months after she first noticed the mass, she presented to our department for a second opinion. She had already consulted over 10 doctors in Romania and Hungary. An excisional biopsy was performed for the 40 × 30 mm IMR mass at another center. No clinical staging was performed, as the presumptive diagnosis then was fibro-lipoma. No imagistic procedures were performed prior to the first surgery.

### 2.2. Imaging

A bilateral mammary MRI was performed. It revealed several lymph nodes (biggest, 12 mm) localized in the right internal quadrant, retro-mammary multifocal lesions, and in the inferior quadrant totalizing, a mass of 35 × 15 mm, without proper demarcation from the pectoralis major muscle and the inter-mammary tissue with infiltration. The right axilla was incompletely visible. The left breast and axilla had a normal appearance. A PET scan was conducted which revealed a 10 × 5 mm right internal mammary lymph node with low uptake, multiple right axillary lymph nodes (biggest 12 mm) with moderate uptake, and a right paramedian epigastric lesion of 16 × 5 mm with low uptake.

**Figure 1:**
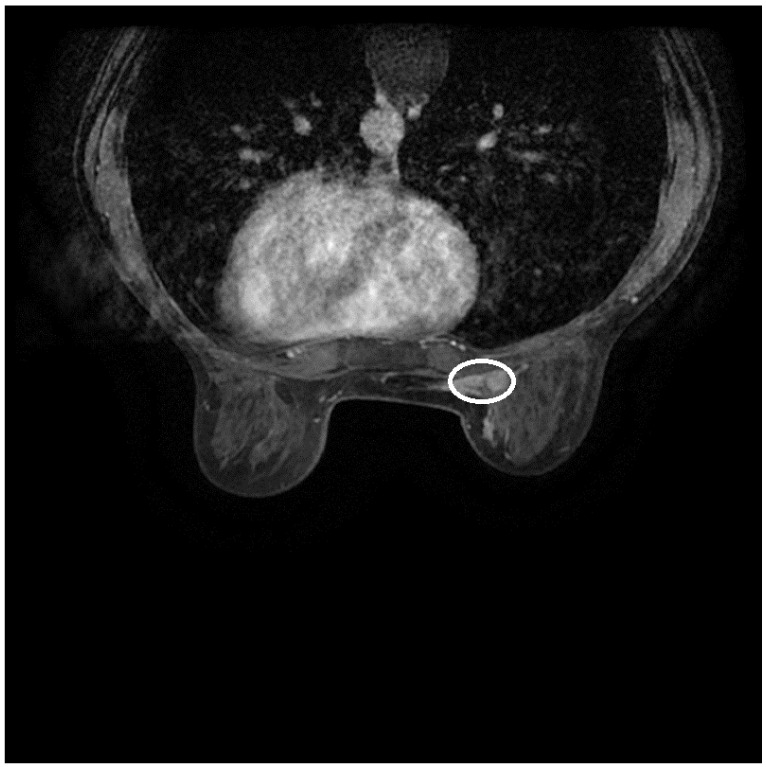
MRI of right breast tumor. Contrast uptake at the level of the right breast tumor.

Bilateral mammography was performed. It showed 8 to 10 pleomorphic micro-calcifications in the internal quadrants of the right breast, with normal appearance of the left breast and both axillae. It suggested BIRADS 3 for the left breast and BIRADS 5 for the right breast (Figure 1).

### 2.3. Classification

**Figure 2:**
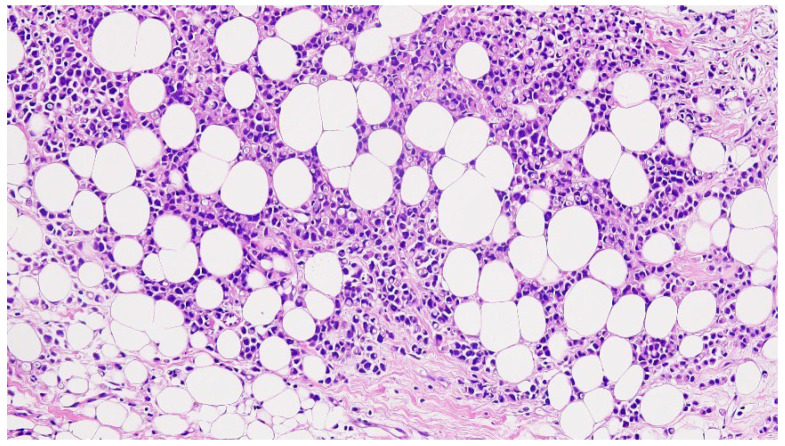
Histopathological findings of invasive lobular carcinoma in infra-mammary region (hematoxylin-eosin staining, 200×).

**Figure 3:**
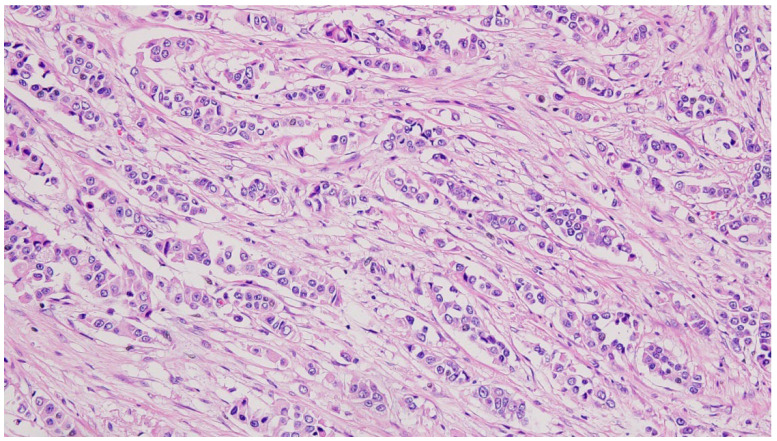
Histopathological findings of non-specific type (NST) G2 ductal invasive grade breast carcinoma (hematoxylin-eosin staining, 200×).

An ultrasound-guided core needle biopsy of the right breast was performed, followed by that of the right axilla one month later. The pathological examination, including immunochemical staining, revealed invasive lobular carcinoma arising from the accessory breast tissue. There was lymphovascular infiltration, and the surgical margins were invaded by tumor. The tumor turned out to be estrogen and progesterone receptor-positive, i.e., ER+, PR+, and Her2/neu-negative, and had a Ki-67 labeling index of 40%. The pathological classification was pT3Pn1R1NxMx. Pathological results showed non-specific type (NST) G2 ductal invasive grade breast carcinoma in the right breast and infiltrative lobular mammary carcinoma with perineural invasion and a similar imunohistochemical profile with the primary IMR tumor (ER-98%, PR-85%, HER2-NEG, KI67-50%). Our tumor board initially categorized the tumors as cT3N1M0 and pT3N1M0 for the primary right IMR accessory breast cancer, stage IIIA, and cT2N0M0 for the right native breast (Figure 2 and Figure 3).

### 2.4. Management

Neoadjuvant chemotherapy comprised Epirubicin, Cyclophosphamide, and Paclitaxel. From March 2023 until September 2023, 4 cycles of Epirubicin (100 mg/m^2^) and Cyclophosphamide (600 mg/m^2^) were given followed by 12 weekly cycles of Paclitaxel (80 mg/m^2^).

**Figure 4:**
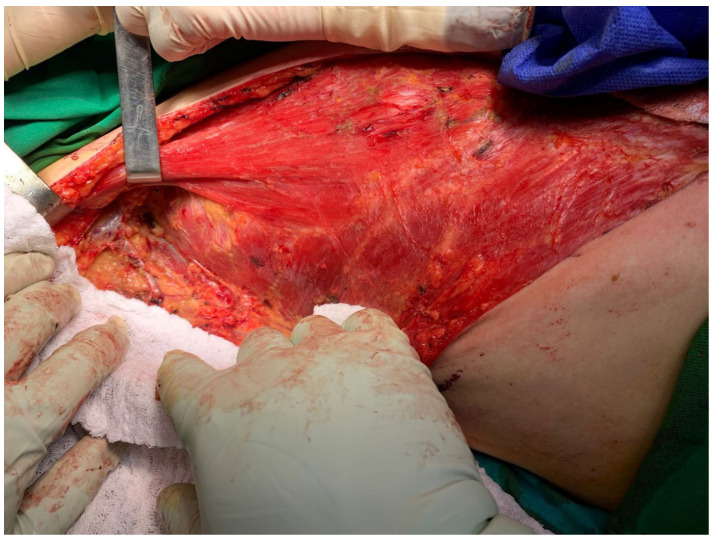
Intra-operative findings. The intraoperative appearance after the excision of the right submammary region and the Madden-type modified radical mastectomy.

**Figure 5:**
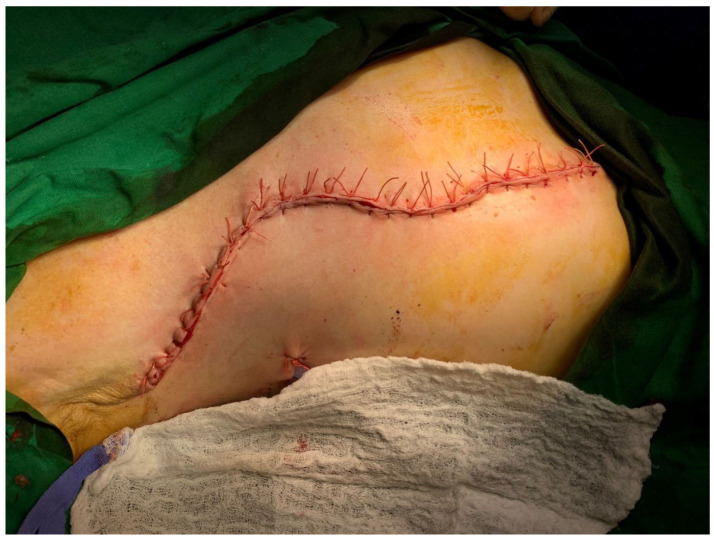
Surgical closure after wide excision of the right submamary region and the Madden-type modified radical mastectomy.

Post-chemotherapy, ultrasound revealed no echographically identifiable tumors in the right breast, right axial, and epigastric regions. The tumor board decided to go ahead with a wide resection right radical mastectomy. Figure 4 and Figure 5 show the intra-operative findings and surgical closure, respectively. Pathological analyses confirmed mammary infiltrative carcinoma NST with residual tissue in the right axilla with carcinomatous metastasis ypR2T2N1aLV0Pn0.

Post-operatively, this patient received adjuvant radiotherapy. Adjuvant radiotherapy was given in the form of 25 fractions of 50 GY, i.e., the patient received 2 GY per day for 25 days. It was given daily on the weekdays, followed by a break on weekends (Saturday and Sunday), resuming on Monday for a total of 25 shots of 2 GY each.

Currently, seven months post-surgery, the patient is recovering well and has been on a Tablet Letrozole 2.5 mg/day dose for the past 5 months. Adjuvant hormonal therapy is to be given for a period of 5 years. Recently, a CT scan was performed and confirmed no signs of relapse.

## 3. Discussion

A small number of primary accessory breast cancer cases have been reported in the currently available literature. Zhang et al. [13] found that 94.7% of cases were females and 5.3% were males. The most common site is the axilla for primary breast carcinoma in the accessory breast tissue [11,14]. According to Evans and Guyton [15], 71% of primary accessory breast cancer cases originate in the axilla. According to Marshall et al., [16] 58% of primary accessory breast cancer cases are found in the axilla, followed by 18.5% parasternal, 8.6% sub-clavicular, 8.6% sub-mammary, and 4% vulvar. The incidence of accessory breast cancer has been reported to be as low as 0.3–0.6% and as high as 6% [17,18,19]. A palpable lump along the milk line, thickened skin, or edema with or without tenderness are common presentations [9,14], which should always raise some degree of suspicion for the presence of ABT and a possibility of its cancer. Important differential diagnoses for ABT are lipoma, lymphadenopathy, sebaceous cyst, vascular malformation, and malignancy [20,21,22].

ABT may have a greater tendency to develop into a malignancy when compared with normal breast tissue due to “stagnation arising in the ductal lumens” [23].

The radiographic findings of accessory breast cancer in breast ultrasound or mammogram are in agreement with those of native breast cancer. Moreover, the management of accessory breast cancer is also similar to that of native breast carcinoma. Breast conservative surgery, along with radiotherapy or mastectomy, remains the standard of care in early-stage breast cancer, with or without adjuvant systemic therapeutic administration, which relies on the status of lymph nodes, hormone receptors, as well as human epidermal growth factor receptor-2 (HER-2) [12]. As an example, breast cancer cells with high HER-2 levels (known as HER-2 positive) have a tendency to grow and spread quickly, but they also respond better to HER-2 protein-targeted therapy, e.g., anti-HER-2 monoclonal antibodies.

In non-metastatic breast cancer, the resection of the tumor along with the adjacent skin, tissue, and regional lymph nodes is the preferred choice [24]. However, pre-operative systemic chemotherapy helps in lowering breast volume and probably lowering the requirement of axillary lymph node dissection (ALND) [24]. Hence, patients having locally advanced pathology may benefit from adjuvant chemotherapy and targeted radiation therapy. In order to alleviate the unwanted effects of radical resection, including large skin defects, reconstructive surgery is preferred [25,26].

In metastatic breast cancer, systemic treatment is favored, with an emphasis on enhancing the quality of life and overall patient survival, and surgery is preferred in selected metastatic cases for palliative therapy [27]. Disease staging, aggressiveness, the patient’s age, and biologic status dictate the multi-modal therapeutic strategy for aggressive breast cancer. Ultrasound findings can be extremely helpful [28,29].

If patients that have already been diagnosed with native breast cancer and have been treated conservatively later present with accessory breast cancer, they may benefit from targeted radiation therapy if previous radiation therapy had not involved the site of accessory breast cancer. Our patient was initially surgically treated for lipoma. The surprising pathological result and the inconsistent attitude of the patient resulted in her visiting over 10 different clinicians in Romania and Hungary, undergoing several imagistic and diagnostic procedures in a chaotic manner over a span of 14 months before a definitive oncologic management plan could be initiated. In our case, accessory breast cancer was associated with synchronous NST carcinoma of the ipsilateral mammary gland. It is not recommended to provide radiation therapy to healthy non-cancerous ipsilateral native breast tissue [18]. However, in the case of additional pathology in ipsilateral native breast tissue and the involvement of axillary lymph nodes, as was the case with our patient, the standard management plan for primary native breast cancer has to be implemented [26,30]. Hence, we went ahead with the recommendation of radiation therapy for site 1, right infra-mammary region, as well as for site 2, right native mammary region, along with right axilla. We provided neoadjuvant chemotherapy with its regimen as described earlier, followed by a wide resection right radical mastectomy. We also provided post-operative adjuvant radiotherapy to this patient with 50 GY at the level of the ipsilateral thoracic wall and axilla. External-beam radiotherapy was given by linear accelerators via sophisticated technologies, including intensity-modulated radiation therapy (IMRT) and image-guided radiation therapy (IGRT), in order to deliver the dose accurately. Various randomized controlled trials available in the current literature utilize different techniques for the delivery of accelerated partial breast irradiation, and this makes a cross-trial comparison difficult [31]. However, without doubt, the use of adjuvant radiotherapy in breast cancer management has resulted in up to 69% reduction in relative risk in loco-regional recurrence at 10 years [32].

The currently established chemotherapy protocol includes Taxane, and it may or may not include anthracycline; however, anthracyclines have been deemed effective in patients at a higher risk, such as those with HER-2-positive and triple-negative subtypes [33]. Our patient was treated with Epirubicin (an anthracycline) and Paclitaxel (Taxane), in line with the latest evidence-based chemotherapy guidelines. The effectiveness of endocrine therapy depends on the tumor’s hormone receptor status [33]. Our patient’s tumor was ER+, PR+, and Her2/neu-negative. Blocking estrogen supply to ER+ breast cancer cells helps in tumor regression [34,35]. Our patient was placed on Letrozole after surgery. Aromatase inhibitors monotherapy (such as Letrozole) has shown better results in terms of the 5-year mortality rate in comparison with Tamoxifen and is favored in high-risk patients and patients with lobular histology [36]. Letrozole has also demonstrated disease-free and distant disease-free survival [37].

It is a challenge to determine the prognosis of patients having accessory breast cancer due to the limited number of documented cases. It has been reported that accessory breast cancers may have poorer outcomes when compared with native breast cancers, but this may be because of the delayed diagnosis of accessory breast cancers [38,39]. Although accessory breast cancer may have a worse prognosis, the lack of long-term follow-up studies invalidates any such predictions [15,40]. Another reason for the poorer outcomes could be that accessory breast cancers have greater involvement of lymph nodes compared to primary native breast carcinomas [41,42]. In our case, despite being in the infra-mammary region, the accessory breast cancer developed separately from the native breast tissue, which had quickly spread to axillary lymph nodes. In this context, a new question raised is as follows: how influential is the role of the histological subtype and the Ki-67 labeling in predicting the metastasis in axillary lymph nodes when we have two different categories of breast cancers at different anatomical locations. More studies should be conducted to address this question.

## 4. Conclusions

To conclude, our case is of special interest because of the following factors:

(i). It is rare to find accessory breast cancer in the infra-mammary region;
(ii). The diagnosis of invasive ductal carcinoma and its classification as cT3N1M0 and pT3N1M0 for the primary right IMR accessory breast cancer, stage IIIA, and cT2N0M0 of the native right breast cancer;
(iii). The timely identification and management involving a multi-modal approach that led to a successful patient outcome for a rare carcinoma that presumably may have had poorer outcomes.

Any cutaneous and/or subcutaneous lesion along the mammary ridge from the axilla to the groin, apart from the native breast tissue, may be accessory breast tissue carcinoma. It should be investigated with suspicion owing to its potential for malignancy. Appropriate imaging and pathological examinations lead to the timely and definitive diagnosis and treatment of accessory breast cancer without delay. Clinically palpable abnormalities and skin changes along the mammary ridge should prompt the consideration of primary breast carcinoma in accessory breast tissue. Early recognition by doctors is necessary to prevent delayed diagnosis and unnecessarily extensive therapy. In our case, the cancer developed from congenital accessory breast tissue. Its timely identification and management involving a multi-modal approach led to a successful patient outcome for this rare carcinoma.

## Data Availability

All the data will be made available upon written valid request.
